# What Is Known About Athlete Engagement: A Scoping Review

**DOI:** 10.1186/s40798-026-00975-5

**Published:** 2026-01-28

**Authors:** Cristina De Francisco, María Claudia Scurtu, M. Pilar Vílchez

**Affiliations:** 1 Universidad de Sevilla, Sevilla, Spain; 2 Universidad Católica de Murcia, Murcia, Spain

**Keywords:** Athlete engagement, Psychological factors, Burnout, Motivation, Perfectionism, Coach-athlete relationship, Athlete engagement questionnaire (AEQ), Sport psychology, Scoping review

## Abstract

**Background:**

Athlete engagement represents a critical construct for understanding optimal athletic functioning, yet the field lacks comprehensive theoretical models integrating its diverse correlates. While previous research has identified various factors influencing engagement, no systematic synthesis exists mapping all empirical evidence across populations, languages, and sporting contexts. This scoping review addresses this critical gap by providing the first comprehensive, systematic synthesis of all factors associated with athlete engagement, establishing the foundational evidence base necessary for theoretical model development and evidence-based interventions.

**Methods:**

The review follows the Preferred Reporting Items for Systematic Reviews and Meta-Analyses extension for Scoping Reviews guidelines, Arksey and O’Malley’s five stages and the Joanna Briggs Institute methodology for scoping reviews. A comprehensive search was carried out in seven databases and 1428 papers were retrieved. After removing duplicates and applying eligibility criteria, 70 studies were assessed for eligibility. After removing papers of poor quality, unavailable papers and papers investigating other types of engagement, 48 papers published between 2007 and 2024 were selected for analysis.

**Results:**

The majority of these papers were quantitative and cross-sectional, using the Athlete Engagement Questionnaire to measure athlete engagement. The review identified 41 correlates of athlete engagement, categorised as antecedents, consequences, mediators or related variables. Psychological factors, such as burnout, motivation, perfectionism and the coach-athlete relationship, were the most commonly studied.

**Conclusions:**

The results highlight the significant role of burnout, motivation, perfectionism and the coach-athlete relationship in enhancing engagement and emphasise the importance of developing training programmes that address these factors in order to promote athlete engagement.

*Registration*: The following scoping review was appropriately registered on the Open Science Framework (10.17605/OSF.IO/TWNZM).

## Key Points

This scoping review analysed athlete engagement from all publications up to May 2024, in all languages and from all sporting backgrounds, in athletes from the age of 12.

The AEQ was the most commonly used tool to measure athlete engagement and Burnout was the most studied variable in relation with athlete engagement.

Forty-one variables were correlated with athlete engagement: 5 demographic variables, 3 sport-related factors (including performance, which was examined as an antecedent, outcome, and mediator), and a further 33 of a psychological nature.

## Background

Athlete engagement has emerged as an important construct for understanding optimal functioning in sport within the framework of positive psychology. While burnout syndrome represents a maladaptive response to sport demands, engagement reflects successful adaptation and thriving in the sport environment [1]. Therefore, athlete engagement is considered a cognitive-affective construct that, although negatively related to burnout, represents a distinct and valuable area of study [2].

Both burnout and engagement constructs originated in occupational psychology and were subsequently adapted to sport contexts [1]. Focusing on engagement specifically, this construct was adapted to sport as a cognitive-affective state composed of four related dimensions: vigour, confidence, dedication, and enthusiasm [3, 4]. Vigour refers to high levels of physical, mental, and emotional energy. Confidence represents the belief in one’s ability to perform at a high level. Dedication involves the desire to invest time and effort to accomplish goals. Finally, enthusiasm is characterized by feelings of excitement and high levels of enjoyment. This definition emerged from the aforementioned studies by the Lonsdale research team [3, 4], the aims of which were: (1) to investigate whether or not athletes experience employee engagement in the work context and, if so, what the dimensions of this engagement were, and (2) to develop a measure of athlete engagement, the Athlete Engagement Questionnaire (AEQ). Although the AEQ was the first instrument to measure athlete engagement, it is not the only one. The Sport Engagement Scale (SES) is an adaptation of the Utrecht Work Engagement Scale, which was originally designed to assess engagement in work settings through three dimensions: absorption, dedication, and vigour [5]. The SES measures these same components in the sports context [5]. Recently, another approach was proposed, by introducing the Sport Engagement Instrument (SpEI) validated with Finnish adolescent athletes with dual careers [6]. The SpEI presents a two-dimensional structure: affective and cognitive, with four factors to measure the affective (i.e., maternal, paternal, coach, and peer social support) and two factors for the cognitive (i.e., control and relevance of sports, and future aspirations and goals) dimensions.

No comprehensive models of athlete engagement have been developed that include key antecedents, potential mediators and moderators, and consequences. Research has been primarily drawn from the field of work engagement, examining antecedent variables such as motivation, gratitude, coping style, social support, care understanding, and coach leadership behaviour [7]. Additional variables studied include basic psychological needs [8–10], perfectionism [10, 11], athlete identity [12], team strength use and individual strength use [13]. Although research on athlete engagement has expanded in recent years, it remains conceptually fragmented and lacks a coherent theoretical framework. Most studies investigate isolated variables, limiting our understanding of how these factors interact to shape engagement. This theoretical gap hinders the development of evidence-based interventions and leaves practitioners without clear guidance on strategies to both foster engagement and prevent burnout. To date, only one review on athlete engagement has been conducted [14], focusing exclusively on elite athletes and drawing from four databases: (Web of Science, Scopus, CNKI, Wanfang Data) in Chinese and English, covering publications from 2001 to 2022.

In contrast, the present study aims to synthesise empirical evidence on the relationship between any variable and athlete engagement—or its subcomponents— by expanding the searchable databases, including all languages, removing publication date restrictions and focusing on athletes aged 12 years and older at any competitive level. A scoping review is the most appropriate approach for this study because of its ability to encompass a wide range of literature on engagement, identifying gaps in knowledge, clarifying concepts, and providing a solid foundation for future research in this area [15]. To enhance transparency and replicability, this scoping review was guided by the PECOS framework. While not commonly applied in scoping reviews, PECOS was chosen to structure a more systematic and rigorous search strategy. Derived from the PICOS model, PECOS replaces the ‘Intervention’ component with ‘Exposure’, as our focus is not on intervention effectiveness but rather on identifying associations between variables [16, 17]. Our research question was “In athletes (P), which factors (E) in the sports context of performance (C) are related to engagement (O) according to empirical evidence (S)?“.

## Methods

This scoping review was designed according to the Preferred Reporting Items for Systematic Reviews and Meta-Analyses extension for Scoping Reviews (PRISMA-ScR) statement [18] and the five stages of Arksey and O’Malley [19]: identifying the research question, searching for relevant studies, selecting studies, mapping data, and collating, synthesising and reporting results.

A protocol was explicitly designed to guide this review and was registered with the Open Science Framework in February 2024 (10.17605/OSF.IO/TWNZM). The proposed scoping review has been conducted following the Joanna Briggs Institute (JBI) methodology for scoping reviews [20].

### Study Selection Criteria

Until May of 2024, two independent reviewers were responsible for searching for relevant studies on the research topic: athlete engagement. Using an advanced search method, papers were collected from the electronic databases EBSCO, Web of Science, Scopus, APA PsycINFO, SPORTDiscus, ScienceDirect, and ERIC in a RIS format. These databases were selected to ensure both comprehensive and targeted coverage of the literature. Web of Science and Scopus provided broad, multidisciplinary reach, while APA PsycINFO and SPORTDiscus ensured depth in psychology and sport-specific research. ScienceDirect and ERIC were included for their relevance to applied social sciences and education, respectively. EBSCO was used as a gateway to access multiple relevant sources, enhancing the overall coverage while avoiding redundancy. In addition to this search, one expert was asked to identify important articles on the topic.

In general, studies were eligible if they reported on research that aimed to relate variables in athletes to sport engagement. Other types of engagement, such as student engagement, media engagement, or community engagement, for example, were excluded. Papers focusing on specific medical conditions or diagnoses were also excluded. This decision was based on preliminary searches, which revealed a high volume of clinically focused studies that did not align with the scope and objectives of the review. In addition, studies involving participants under the age of 12 were excluded, as sport engagement at this developmental stage is typically characterized by *deliberate play* rather than *deliberate practice*, and is largely unstructured and parent-initiated [21]. According to the Developmental Model of Sport Participation, this period corresponds to the *sampling years*, during which children are exposed to various sports in playful settings, with limited relevance to the more autonomous, sustained engagement observed in adolescence and beyond. Therefore, the review focused on youth and adult populations, from early adolescence onward, when individuals begin to engage more purposefully and independently in sport. Furthermore, setting the lower age limit at 12 years ensured that participants had the cognitive maturity necessary to adequately understand and respond to the questionnaires used in the studies. Moreover, the term ‘federated athlete’ refers to individuals officially registered with a recognized sports federation or governing body, which entails formal participation in structured training and regulated competitions through a club or association. Table 1 shows the eligibility criteria of papers for this scoping review.

### Search Strategy and Process

Records from the database were exported to Rayyan software [22]. This is a collaborative web application designed to enhance collaboration, streamline workflow, and ensure rigour and reproducibility of systematic reviews, providing a comprehensive solution from papers import to screening, data extraction, and analysis. A total of 1428 papers were retrieved from databases and imported into Rayyan as follows: EBSCO (456), Web of Science (344), Scopus (337), APA PsycINFO (125), SPORTDiscus (86), ScienceDirect (40), and ERIC (40). Duplicate articles (827) were removed, and a sports research specialist recommended two more papers. Two reviewers then proceeded independently to screen the titles and abstracts of the remained 603 papers. In this process, Rayyan’s keyword-based exclusion functionality was used, and additional keywords emerged inductively through engagement with the material during screening. These emergent terms—such as *work engagement*, *social media engagement*, *student engagement*, *consumer engagement*, *community engagement*, *volunteer engagement*, among others—were identified as recurring and relevant within the dataset. This approach allowed us to refine the scope of the review based on the actual thematic focus of the literature, enhancing transparency and replicability.

### Data Extraction Procedure

Two reviewers independently screened the remaining 603 records. Titles and abstracts were reviewed for relevance based on predefined inclusion criteria (Table 1).

**Table 1:** Eligibility criteria

Description	Search terms
Engagement	“engagement AND athlet*” [title/abstract] OR “engagement AND sport*” [title/abstract]
Population	Athletes
Type of athletes	Federated athletes who compete
Age	Over 12 years old
Date	Without date limit
Publication type	Empirical studies or case studies (Systematic reviews, Meta-analyses, theoretical papers, dissertations and conference abstracts, interventional studies were excluded)
Language	All languages

**Fig. 1:**
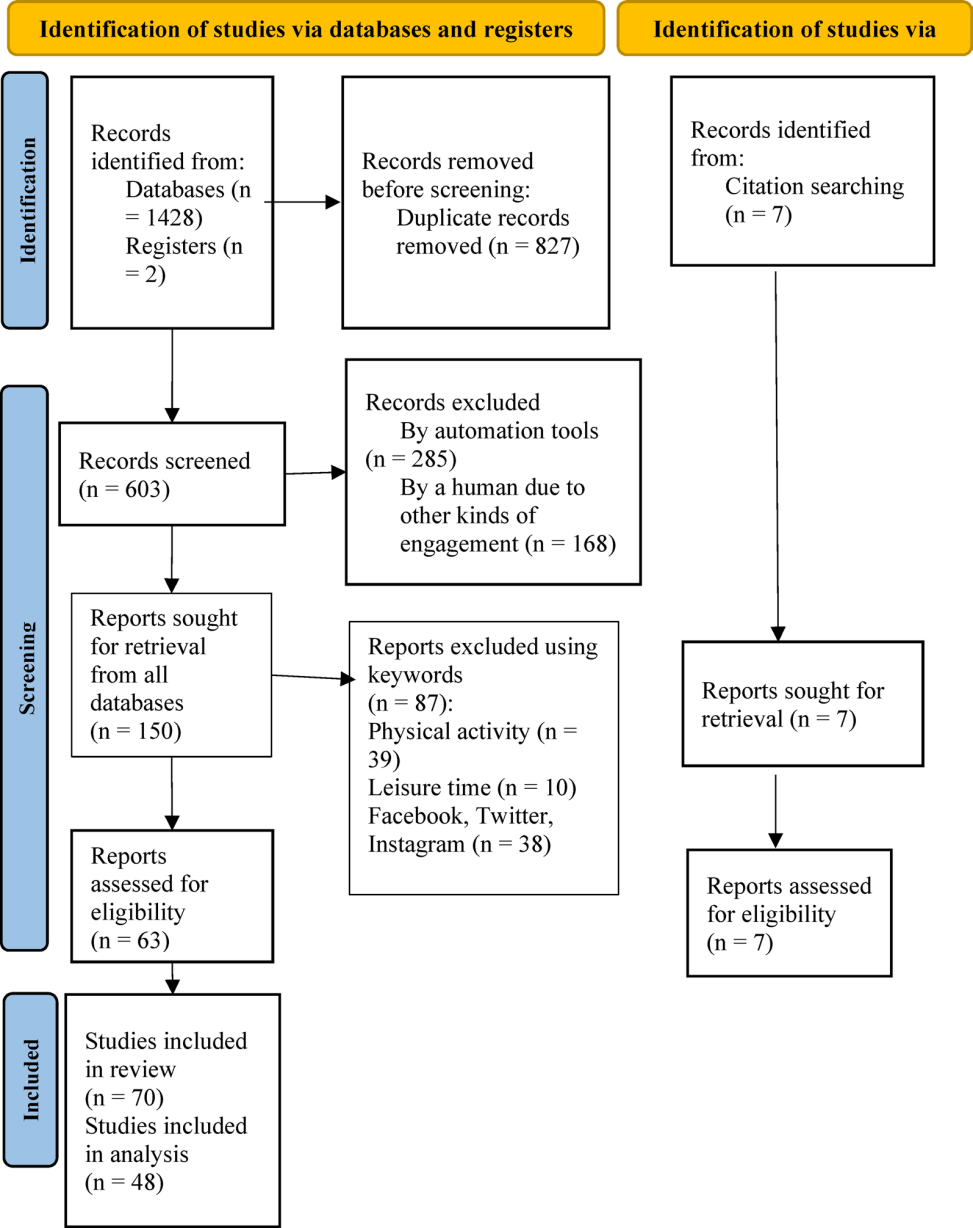
PRISMA 2020 flow diagram [27]

Following this step, full texts of potentially eligible articles were assessed for inclusion. An Excel spreadsheet of all 70 eligible citations (63 selected from databases and 7 records from external sources) was then created following the JBI Manual for Evidence Synthesis [23]. The spreadsheet included the following information for each citation: author(s), year of publication, origin/country of origin, aims/objectives, population/sample size, methodology/methods, study/intervention type, and key findings related to the scoping review question. The complete selection process is summarised in Fig. 1, in accordance with the PRISMA-ScR flow diagram structure.

### Quality Assessment and Risk of Bias

While critical appraisal is not typically required in scoping reviews, a basic assessment of methodological quality was conducted to enhance the interpretability of the findings and to provide readers with an overall indication of the robustness of the included studies. To assess the methodological quality of the studies included in this review, the Spanish version of the PEDro scale was used [24]. It consists of 11 items related to specified eligibility criteria, random allocation, concealed allocation, similarity at baseline, blinding of subjects, blinding of therapists, blinding of assessors, > 85% follow-up for at least one key outcome, intention-to-treat analysis, statistical comparison between groups for at least one key outcome, and point and variability measures for at least one key outcome. Any discrepancies were resolved by consensus or by referring the matter to a third researcher. Items that scored at least 8 out of 11 possible points on the PEDro scale were considered acceptable, as randomised controlled trials (RCTs) [25, 26].

## Results

The complete selection process is summarised in Fig. 1, in accordance with the PRISMA-ScR flow diagram structure.

**Fig. 2:**
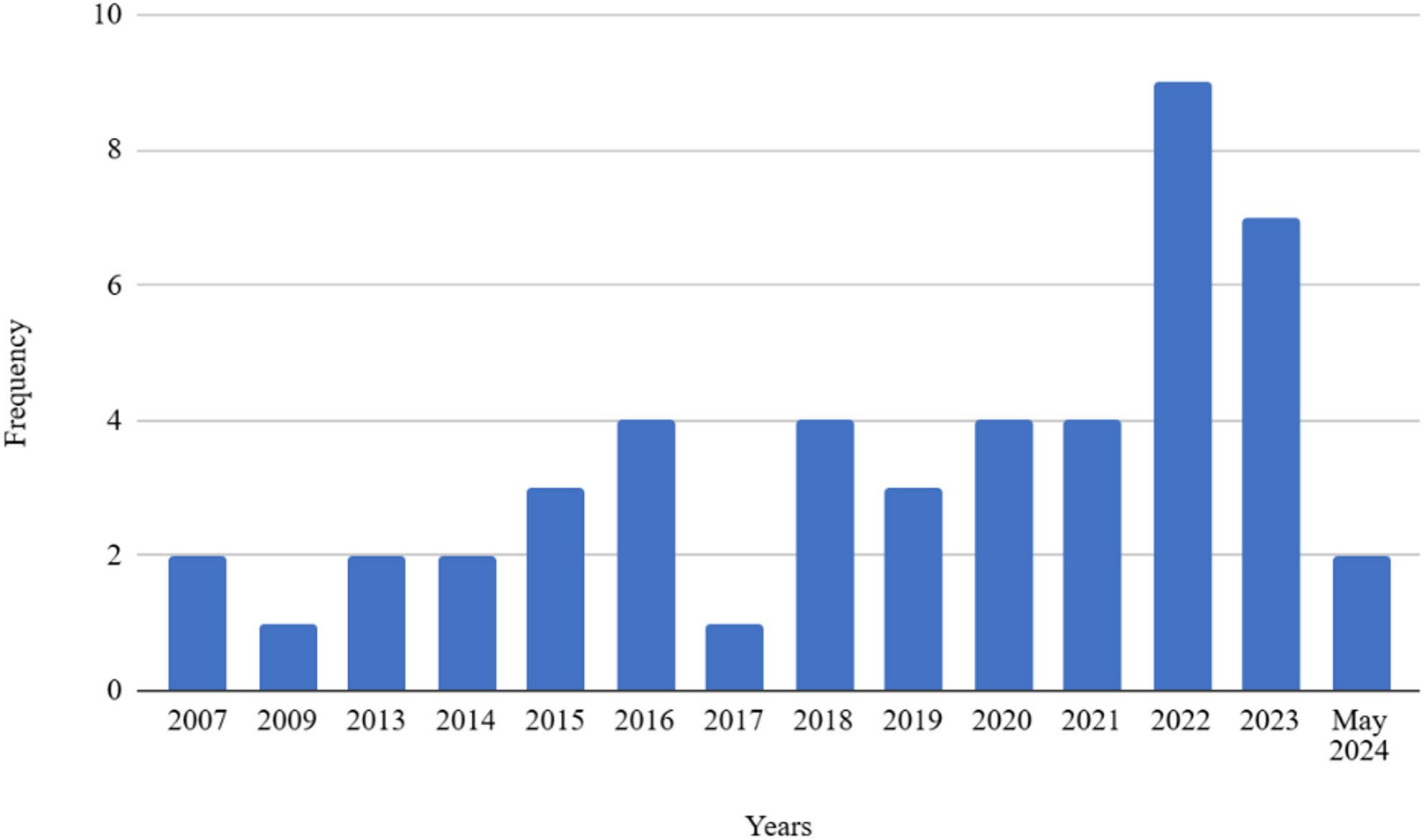
Years of publication of included studies

A total of 48 studies were selected (Table 2). A quantitative approach was adopted in the majority of studies included in the review, with only one qualitative investigation. Of the studies identified, six were longitudinal and forty-two were cross-sectional. The publication years of the selected articles spanned the period from 2007 to May 2024 (until data collection was completed; see Fig. 2). The number of publications per year has generally increased over time.

**Table 2:** Studies included in the review

Study	Objectives	Participants	Instruments	Findings
Aykin and Ermisket 2022 [28]	Examine task and ego orientations and levels of engagement in sport according to athletes’ perceptions of coaches’ leadership styles.	310 Turkish basketball players	Turkish versions of: Task and Ego Orientation in Sports Questionnaire (TEOSQ) [29]. Sports Engagement Scale (SES) [30].	Task and ego orientation and sport engagement are higher among women and secondary school graduates. Those who have played basketball for 3–4 years have a higher level of sports involvement. Those with a family income of 7501 TL and above have a higher level of involvement and those who live in the district have a lower level of involvement. The task and ego orientation levels of athletes with charismatic coaches were higher than those with democratic, authoritarian and liberal coaches.
Babić et al. 2015 [12]	Determine the construct validity and reliability of the AEQ and AIMS and differences in their dimensions according to sex, educational level and medal winning in international competitions.	71 Croatian sprinters	Croatian versions of: Athlete Engagement Questionnaire (AEQ) [12]. Athletic Identity Measurement Scale (AIMS) [12].	The sport engagement and sport identity dimensions were statistically significantly correlated, particularly among male sprinters. Females scored higher on identity/exclusivity and males scored higher on self-identity. Athletes with international medals were more enthusiastic about sport engagement and no significant differences were found in sport identity. Educational level had no significant effect on sport engagement or sport identity dimensions.
Cahyono et al. 2023 [31]	Examine the relationship between coach leadership style (CLS), coach-athlete relationships (CAR), athlete burnout (AB) and athlete engagement (AE) in team sports.	93 Indonesian athletes in team sports	Indonesian versions of: Coach Leadership Scale for Sport (CLSS) [31]. Coach–Athlete Relationship Questionnaire (CART-Q) [31]. Athlete Burnout Questionnaire (ABQ) [31]. AEQ [31].	Coach leadership style had a direct influence on coach-athlete relationship, athlete burnout and athlete engagement. Highlights: The important role of coach leadership style and coach-athlete relationship in reducing the risk of athlete burnout and improving athlete engagement in team sports.
Chao et al. 2016 [32]	Examine the relationship in a moderated mediation model where athletes’ subjective well-being mediates the relationship between sport mental toughness and athlete engagement, and assess how this mediation effect is moderated by negative physical self-perception.	232 Chinese volleyball players	Sports Mental Toughness Questionnaire (SMTQ) [33]. AEQ [4]. Subjective Well-Being Scale for Chinese Athletes (SWBS) [34]. Negative Physical Self Scale (NPSS-G) [35].	Volleyball players’ subjective well-being has a partial mediating effect between sport mental toughness and athlete engagement. Sport mental toughness has a direct influence on athlete engagement and promotes athlete engagement indirectly by increasing the score of volleyball players’ subjective well-being. Negative physical self moderates this mediation effect. When negative physical self increases, the effect of Chinese volleyball players’ subjective well-being on athlete engagement decreases.
Curran et al. 2015 [36]	Understand the coach behaviours that promote positive experiences in youth sport by examining the relationships between motivational climate and athlete engagement (i.e. confidence, dedication, enthusiasm and vigour).	260 British soccer players	Perceived Motivational Climate in Sport Questionnaire-2 (PMCSQ-2) [37]. AEQ [4].	Athlete engagement was positively predicted by a mastery climate. Cognitive aspects of athlete engagement were positively predicted by a mastery climate. The motivational climate is associated with engagement and a mastery climate and offers a means of promoting higher levels of overall engagement.
De Francisco et al. 2017 [38]	Adapt the Athlete Engagement Questionnaire to Spanish context.	509 Spanish athletes	Spanish version of AEQ [38].	All estimated parameters were statistically significant and the overall fit of the model was reasonable. Cronbach’s alpha values were also satisfactory for each factor, with values above the 0.70 cut-off. The Spanish version of the AEQ offers psychometric properties similar to those found in the original version.
De Francisco et al. 2018 [39]	Explore the role of self-motivation as a mediator between the satisfaction of basic psychological needs and athlete engagement	1157 Spanish athletes	Spanish versions of: Basic Needs Satisfaction in Sport Scale (BNSSS) [40]. Behavioral Regulation in Sport Questionnaire (BRSQ) [41]. AEQ [38].	High levels of self-motivation increase athlete engagement and vice versa. - Basic needs satisfaction is positively related to the most self-determined types of motivation and negatively related to the least self-determined types. Motivation plays a mediating role between needs satisfaction and engagement
De Francisco et al. 2018 [42]	Analyse the measurement invariance of the Athlete Engagement Questionnaire across levels of competition and provide evidence of construct validity in relation to burnout.	426 Spanish athletes	Spanish versions of: AEQ [38]. ABQ [43].	The invariance of the AEQ between athletes participating at different levels of competition. The four engagement dimensions correlated negatively with the three burnout dimensions.
De Francisco et al. 2020 [8]	Evaluate the mediating role of motivational regulation between the satisfaction of basic psychological needs and burnout and engagement in athletes.	1011 Spanish athletes	Spanish versions of: BNSSS[40]. BRSQ [41]. Short version of ABQ [44]. AEQ [38].	The satisfaction of basic psychological needs (BPN) had a negative direct effect on sport burnout and a positive direct effect on engagement, and an indirect effect through two mediating variables: low and high self-determined motivation. Satisfaction of BPN is positively related to high self-determined motivation and negatively related to low self-determined motivation. There was a positive and moderate relationship between low levels of self-determined motivation and burnout. The most self-determined levels had a positive effect on sport engagement, explaining 21% of its variance.
DeFreese and Smith 2013 [1]	Investigate the nature of the relationship between burnout and engagement in sport using comparative models that incorporate work life areas.	210 American football players	Areas of Worklife Survey adapted to sport (AWLS) [1]. ABQ [45, 46]. AEQ [4].	Moderate scores on the work life variables, with the exception of community, which was high on average relative to the response set options. Low levels of the athlete burnout dimensions of emotional and physical exhaustion, reduced sense of accomplishment and sport devaluation relative to the response set options. Higher workload scores on the AWLS represent greater workload manageability. Negative correlations of workload with burnout dimensions and positive correlations of workload with engagement dimensions. The comparison of two structural models provided preliminary evidence that athlete burnout and athlete engagement may not lie on a global continuum. The global areas of worklife variable used in the structural models strongly predicted both burnout and engagement.
Demirdoken et al. 2019 [47]	Characterize the degree of engagement among students of the Faculty of Sport.	834 Turkish students athletes	Turkish version of AEQ [48].	Significant difference in the dimension ‘Enthusiasm’ according to departments for female students. Significant difference in the dimensions of confidence, commitment and enthusiasm for male students. The sub-dimension of robustness for female students according to their status in individual or team sports shows a significant difference, and the sub-dimension of enthusiasm for male students differentiates. According to the age of the sport, there was a significant difference in all subdimensions for female and male students, except for commitment.
Graña et al. 2021 [49]	Analyse the relationship between motivation, burnout and engagement in sport.	500 Spanish athletes	Spanish version of: Sport Motivation Scale (SMS) [50]. AEQ [38]. ABQ [43].	Motivation was negatively related to burnout and positively related to engagement. Burnout and engagement are inversely related. Engagement plays a mediating role between motivation and burnout. There are no sex differences in this relationship, and there are no differences between individual and collective sportspeople.
Gu and Xue 2022 [51]	Study the influence of the coach-athlete relationship (CAR) on the engagement of athletes and the mediating effect of the thriving between them.	326 Chinese athletes in team sports	Chinese versions of: Psychological Collectivism Questionnaire (PCQ)[52]. Group Environment Questionnaire (GEQ) [53]. SMTQ [54]. AEQ [55].	The total score of cohesion and all dimensions can significantly and positively predict athlete engagement, among which ATG-T plays an important role in predicting athlete engagement. Cohesion can influence athlete engagement through a direct and an indirect path. The indirect path involves the mediating role of psychological collectivism, the mediating role of mental toughness, and the sequential mediating role of psychological collectivism and mental toughness.
Gu et al. 2023 [7]	Examine the influence of the coach-athlete relationship (CAR) on athlete engagement and evaluate the mediating effect of thriving between them based on self-determination theory.	287 Chinese athletes in team sports	Chinese versions of: CART-Q [56]. Thriving Scale [57]. AEQ [55].	CAR and its dimensions can significantly and positively predict athlete engagement, complementarity, commitment and closeness. The direct and indirect paths show that CAR influences athlete engagement through the mediating effect of thriving. After controlling for sex, age, years of training and sport performance, regression analysis showed that the total CAR score explained 36% of athlete engagement.
Guillén and Martínez-Alvarado 2014 [5]	Adapt the Utrecht Work Engagement Scale (UWES) to a sports environment and to validate the scale with a sample of Spanish soccer players.	240 Spanish soccer players	SES [5]. Inventario de Burnout en Deportistas (IBD) [58].	The Sport Engagement Scale (SES) consists of 13 items divided into three factors: Vigour (4 items), Commitment (4 items) and Absorption (5 items). Construct validity was confirmed by regression analysis with the original UWES. The concurrent validity of the SES scale was confirmed by correlations with known indicators of sport burnout. SES is a reliable and valid instrument for measuring athlete engagement
Guo et al. 2022 [59]	Explore the causal relationship between athlete gratitude, athlete engagement, and athlete burnout through cross-lag analysis of longitudinal associations.	Time 1= 476 Time 2 = 352 Chinese athletes in team sports	Chinese versions of: Gratitude Questionnaire (GQ). [60]. AEQ [61]. ABQ [62].	The overall level of athlete gratitude and engagement was high. Chinese athletes at Master level had higher levels of gratitude and athlete engagement than athletes at I and II levels. Athlete gratitude is a significant negative predictor of athlete burnout and a significant positive predictor of athlete engagement. Cross-lagged analyses indicated a reciprocal relationship, such that earlier engagement predicted later burnout, and earlier burnout predicted later engagement. Athlete gratitude indirectly influences athlete burnout through athlete engagement, and also indirectly influences athlete engagement through athlete burnout.
Guojie 2021 [63]	Examine the impact of athletic psychology and athlete engagement on athletic performance, as well as the influence of athletic performance on the success of athletes at a sports center in China, while also exploring the mediating effects of coaching behavior on these relationships.	290 Chinese athletes	Chinese versions of: Athletic Psychology (APSY) [63]. AEQ [63]. Athlete Performance (AP) [63]. Coaching Behavior (CB) [63]. Athletes’ Sports Success (ASS) [63].	Athletic psychology and athlete engagement have a positive association with athletic performance Athletic performance also has a positive association with athletic success of athletes in the sports centre in China Athletic performance significantly mediates the nexus of sport psychology, athlete engagement and athlete sport success. Coaching behaviour significantly moderates among the nexus of athletic performance and athlete sport success.
Habeeb et al. 2023 [64]	Investigate the direct association of parent, coach, and peer-initiated motivational climate with burnout and engagement in high school athletes, and assess whether peer-initiated motivational climate mediates or moderates the impact of coach-initiated motivational climate on these outcomes	150 American athletes	Parent-Initiated Motivational Climate Questionnaire-2 (PIMCQ-2) [65, 66]. Motivational Climate Scale for Youth Sports [67]. Peer Motivational Climate in Youth Sport Questionnaire [68]. A BQ [46]. AEQ [4].	Results supported the mediation model, but not the moderation model. Significant medium to large indirect pathways from coach mastery climate → peer mastery climate → burnout, and coach mastery climate → peer mastery climate → engagement. Parent, coach and peer mastery motivational climates were associated with burnout and engagement, whereas achievement climates were largely unrelated to these indices of athlete well-being. Coaches also had an indirect association through peer mastery initiated motivational climate.
Hill et al. 2020 [69]	Examine the interactive effects of dimensions of perfectionism in predicting athlete engagement and test the hypotheses of the 2 × 2 model of perfectionism, while also investigating whether support for this model varies depending on the instrument used to measure perfectionism.	Sample 1 = 297 British swimmers Sample 2 = 222 British athletes Sample 3 = 211 British athletes	Multidimensional Perfectionism Scale was adapted to focus on athletes in sport [69]. Multidimensional Inventory of Perfectionism in Sport (MIPS) [70]. Sport-Multidimensional Perfectionism Scale-2 (S-MPS-2) [71, 72]. AEQ [4].	The 2 × 2 model may explain differences between athletes in levels of engagement. These differences will depend on which indicators of PSP and ECP were examined.
Hodge et al. 2009 [9]	Examine the hypothesized antecedents (basic psychological needs) and consequences (dispositional flow) of athlete engagement, and assess the extent to which athlete engagement mediates the relationship between basic psychological needs and flow.	201 Canadian athletes	Basic Needs [9]. AEQ [4]. Dispositional Flow Scale-2 (DFS-2) [73, 74].	Athletes had moderate to high levels of needs satisfaction, athlete engagement (AE) and flow. Global flow was strongly correlated with three of the AE dimensions: confidence, vigour and enthusiasm, while there was a moderate relationship between global flow and dedication. Vigour had a strong association with autotelic experience and a moderate association with challenge-skill balance, clear goals, concentration and sense of control. Enthusiasm had a strong positive relationship with autotelic experience, and moderate positive relationships with sense of control and clear goals. Dedication had moderate positive relationships with clear goals, autotelic experience and concentration. Confidence had a strong relationship with challenge-skill balance, sense of control, and autotelic experience. The relationships between needs and flow were not fully mediated by AE (competence and autonomy predicted flow, but relatedness did not).
Jaramillo-Tapia et al. 2024 [75]	Analyse the relationship between sport engagement and mental toughness in high level competitive tennis players from Ecuador and Portugal.	265 Portuguese and Ecuadorian tennis players	Mental Toughness Index (MTI) Spanish: [76]; Portuguese [75]. AEQ [42]; Portuguese [77].	Tennis players from both countries have high levels of commitment and mental toughness. A positive relationship was found between sport commitment and mental toughness, suggesting that players need to be dedicated and enthusiastic about the sport in order to become mentally tougher individuals. No significant differences were found between the two sexes and countries.
Jowett et al. 2016 [10]	Examine whether perfectionistic concerns and perfectionistic strivings are differently associated with athlete burnout and engagement, and explore how these associations are explained by basic psychological needs, as guided by self-determination theory.	222 British athletes	ABQ [46]. AEQ [4]. S-MPS-2 [72]. Short version Multidimensional Perfectionism Scale (H-MPS) [78]. BNSSS [79]. Psychological Need Thwarting Scale (PNTS) [80].	Basic psychological need satisfaction and thwarting mediated the perfectionism-engagement and perfectionism-burnout relationships. Perfectionistic concerns shared a negative relationship (via need satisfaction) with athlete engagement and a positive relationship (via need satisfaction and thwarting) with athlete burnout. Perfectionistic striving had a positive relationship with athlete engagement and a negative relationship (with athlete burnout).
Kelecek et al. 2018 [48]	Test the reliability and validity of the Turkish version of the Athlete Engagement Questionnaire.	201 Turkish athletes	Turkish versions of: ABQ [48]. AEQ [48].	The results of principal component factor analysis with varimax rotation showed that four factors explained 71.73% of the total scale. Internal consistency coefficients ranged from 0.75 (dedication) to 0.92 (confidence). The AEQ scores were not significantly correlated with the ABQ. The Turkish version of the AEQ can be used to determine the level of engagement of athletes, but the criterion-related validity is questionable.
Kosmidou et al. 2013 [81]	Examine the relationship between physical self worth, athletic engagement and goal orientation in Greek female athletes.	258 Greek athletes	Greek versions of: Physical Self-Perception Profile (PSPP) [82]. AEQ [83]. TEOSQ [84, 85].	Athletes had similar physical self worth whether they were current or former athletes. Athletes in individual sports had higher physical self worth than athletes in team sports. Physical self worth was predicted by confidence, dedication and ego goal orientation.
Kuokkanen et al. 2021 [6]	Develop the Sport Engagement Instrument (SpEI) within the Finnish dual career context.	Sample 1 = 992 Finnish athletes Sample 2 = 465 Finnish athletes	Sport Engagement Instrument (SpEI) [6].	18 items, along with four affective engagement factors and either two first-order or one second-order cognitive engagement factor best described the phenomenon of sport engagement in both samples. Higher levels of sport burnout correlated negatively and behavioural engagement correlated positively with the affective and cognitive dimensions of engagement, supporting the validity of the instrument.
Limpo et al. 2022 [86]	Compare karate and soccer athletes’ perceptions of the benefits and risks of aggression in their sports, and to test whether these perceptions predict athletes’ engagement and quality of life (QoL).	100 Portuguese karate practitioners and soccer players	Portuguese versions of: Perceived Benefits and Aggressiveness Risks Scale (PBAR Scale) [87]. Utrecht Work Engagement Scale ( UWES) [86]. Europe Health Interview Surveys Quality of Life Abbreviated Instrument (EUROHIS-QOL-8) [88].	Karatekas perceived more benefits and fewer risks in karate than in football, but footballers generally perceived equal benefits and risks in both sports. Both perceived similar physical and psychological benefits in their own sport, but perceived physical benefits as the most important outcome in the other sport. Karatekas perceived benefits of karate predicted engagement directly and QoL indirectly via vigour. Karate athletes’ perceptions appeared to be relevant to the experience of fulfilment in training and general well-being.
Liu et al. 2023 [89]	Examine whether athlete engagement (AE) mediates the relationship between partnership and competitive performance, in line with self-determination theory.	242 Chinese dance sport participants	Chinese versions of: Partnership Scale Dance Sport Couples (PS-DSC) [89]. AEQ [55]. Competitive Performance Questionnaire [89]. Athlete Satisfaction Questionnaire (ASQ) [89].	Obligatory instrumental ties, expressive ties and interpersonal perception scores are all positively correlated with both athlete engagement and competitive performance, and athlete engagement scores are positively correlated with competitive performance. Athlete engagement fully mediates the association between obligatory instrumental ties and competitive performance, and it partially mediates the association between expressive ties, interpersonal perception, and competitive performance.
Lonsdale et al. 2007 [3]	Determine whether elite athletes are engaged and identify the common dimensions of athlete engagement.	15 New Zealand athletes	Interview based on the Scalan Collaborative Interview Method (SCIM) [90].	Athlete engagement was relevant to elite athletes and it is hoped that the results of the study will lead to research into the promotion of positive sport environments.
Lonsdale et al. 2007 [4]	Develop a measure of core athlete engagement dimensions (confidence, dedication, and vigor), investigate whether preoccupation and enjoyment also constitute core dimensions of athlete engagement, and provide evidence of nomological validity by examining associations with athlete burnout scores.	-382 New Zealand athletes -201 New Zealand athletes -343 Canadian athletes	AEQ [4]. ABQ [46].	Enjoyment was strongly related to AE. However, the enjoyment items were not subsumed under the vigour factor. Scores derived from items intended to represent enjoyment and excitement formed a separate factor labelled ‘enthusiasm’. The four-factor Athlete Engagement Questionnaire (AEQ), designed to measure confidence, dedication, vigour and enthusiasm, appeared to best fit the data in Study 1. The factorial validity of scores derived from the AEQ was also supported in Studies 2 and 3. In Study 3, the nomological validity of the AEQ scores was supported by negative correlations between athlete burnout and athlete engagement scores.
Martinent et al. 2021 [91]	Examine whether there are subgroups of athletes with differing levels of basic psychological need (BPN) satisfaction and frustration, and to explore differences in BPN profiles based on sex, years of experience, and number of training hours. Additionally, the study aims to investigate changes in these profiles over time and determine if athletes from different BPN profiles exhibit variations in terms of sport burnout and engagement	367 French athletes	Basic Need Satisfaction in Sporting Context Scale (BNSSCS) [92]. Athlete Burnout Scale (ABO-S) [93]. French version of PNTS [91]. Shortened version of UWES-9 [91].	The results showed three profiles at each time point: fulfilled, autonomy and competence moderately frustrated, and competence and relatedness highly frustrated profiles. Male athletes and athletes with more years of experience were more likely to belong to the autonomy and competence moderately frustrated profile compared to the other BPN profiles. Athletes who trained more hours per week were more likely to belong to the fulfilled profile compared to the autonomy and competence moderately frustrated profile. Individuals in the fulfilled and autonomy and competence moderately frustrated profiles were particularly likely to remain in their respective profiles at Times 2 and 3, whereas individuals in the competence and relatedness highly frustrated profile were less stable in their profile membership. Individuals from the fulfilled profiles reported lower burnout scores and higher engagement scores than those from the other two BPN profiles. The opposite pattern of results was observed for the competence and relatedness highly frustrated profile.
Martínez-Alvarado et al. 2016 [94]	Analyze the relationships between basic motivational needs, burnout, and engagement among soccer players to elucidate why these athletes either remain engaged or experience burnout.	227 Spanish soccer players	Perceived Autonomy Scale [95]. Perceived Competence Scale from the Intrinsic Motivation Questionnaire (IMI) [95]. Need for Relatedness Scale [95]. IBD [58]. Athlete Engagement Scale ( AES) [94].	Footballers reported moderate levels of satisfaction of psychological needs for competence, autonomy and relatedness, and low to moderate levels of athlete burnout symptoms of emotional exhaustion, reduced personal accomplishment and depersonalisation, as well as moderate to low levels of burnout factors on the global assessment. Moderate to high levels were reported for vigour, dedication and absorption. Positive and negative predictions of basic psychological needs for engagement and burnout, respectively. The need for autonomy is the variable that best predicts the symptoms of burnout and engagement in Spanish third division soccer players. Reduced personal accomplishment was the strongest predictor of burnout within the model.
Martins et al. 2014 [77]	Test the reliability and validity of the Portuguese version of the Athlete Engagement Questionnaire.	Sample 1 = 357 Portuguese athletes; Sample 2 = 414 Portuguese athletes	Portuguese version of AEQ [77].	The results of the CFA indicated that the four-factor structure provided a good fit to the data and that all constructs had good psychometric properties.
McGee and DeFreese 2019 [96]	Examine associations between athletes’ perceptions of the coach-athlete relationship and psychological outcomes of burnout and engagement over a competitive season.	37 American rowers	CART-Q [97]. Perceived Stress Scale (PSS) [98]. SMS [99]. ABQ [46]. AEQ [4].	Closeness was a significant predictor of seasonal global burnout and engagement over the seasonal assessment period.
Mellano and Pacewicz 2022 [2]	Examine the interaction of teammate and coach support on athlete burnout and engagement, and to determine how type of teammate and coach support is related to athlete burnout and engagement.	176 American athletes	Perceived Available Support in Sport Questionnaire (PASS-Q) [2]. ABQ [46]. AEQ [4].	Total teammate and coach support accounted for 14.9%-26.0% and 22.4%-36.7% of the variance explained for the dimensions of burnout and engagement, respectively. No significant interactions were found. Teammate esteem support predicted lower achievement, devaluation, confidence and vigour, whereas emotional and tangible support predicted dedication and enthusiasm. - Coach esteem support predicted lower performance and devaluation.
Pedro and Veloso 2018 [100]	Explore, within the framework of self-determination theory, coach autonomy support and athlete engagement and their relationship and contribution to resilience.	177 Portuguese athletes	Portuguese versions of: Autonomy subscale of Basic Psychological Needs Support Perception [100]. AEQ [96]. Short version of Resilience Scale (RS13-A) [101].	Coach autonomy support and athlete engagement are positively associated with resilience, and some dimensions of engagement are more important than others for resilience development.
Podlog et al. 2015 [102]	Examine whether the different types of motivation articulated in self-determination theory mediate the relationship between basic need satisfaction (competence, autonomy, and relatedness) and athlete engagement.	192 Swedish downhill skiers	Swedish versions of: Competence subscale of the Intrinsic Motivation Inventory [102]. Autonomy scale developed by Hollembeak and Amorose [103, 102]. Feelings of Relatedness Scale [102]. Situational Motivation Scale (SIMS) [102]. AEQ [102].	All four motivational regulations were significant partial mediators of the relationship between autonomy support and engagement. With the exception of external regulation, all three motivational regulations fully mediated the relationship between relatedness and engagement. Intrinsic motivation and identified regulation partially mediated the relationship between competence and engagement.
Raimundi et al. 2023 [104]	Examine the perceptions and observed behaviors of athletes and coaches regarding the motivational climate created by the coach during training and matches over the course of a season, and assess whether these sources of information can inform the engagement of young basketball and volleyball players.	517 Argentinian basketball and volleyball players 48 coaches	Spanish version of multidimensional Motivational Climate Observational System (MMCOS) [105]. Argentinian versions of: AEQ [106]. Empowering and Disempowering Motivational Climate Questionnaire-Coach (EDMCQ-C) [107].	Differences were found between the perspectives and, in general, a decrease in variables characterising an empowering climate and an increase in those characterising a disempowering climate were observed over the course of the season. When all measures are considered together and the effect of time is controlled for, the assessments that predict engagement are athlete perceptions and match observations.
Raimundi et al. 2024 [108]	Validate the Argentinian versión of Athlete Engagement Questionnaire in young athletes.	1188 Argentinian athletes	Argentinian version of AEQ [108]. BRSQ [109]. BPNSS [95]. Argentinian version of Sport Enjoyment Scale (SES) [110]. Short version of ABQ [44].	Confirmed the multidimensional nature of engagement, showing positive associations with high quality athlete experiences and confirming the inverse relationship with burnout. Male athletes, younger athletes and those with a higher competitive level showed more engagement and interactions between these socio-demographic variables. The Argentine version of the AEQ showed optimal fit and reliability and good indices of measurement invariance across sex, age and competitive level. Engagement is an indicator of an optimal experience associated with positive youth development through sport participation.
Sarı and Bizan 2022 [111]	Investigate the role of parent-initiated motivational climate on athletes’ dispositional flow and sport engagement.	180 Turkish athletes	Turkish versions of: Parent-Initiated Motivational Climate Questionnaire-2 [112]. AEQ [48]. Short Dispositional Flow Scale-2 (SDFS-2) [113].	Confidence, dedication, vigour, enthusiasm, global engagement and dispositional flow were positively and significantly correlated with parent-initiated task-involving climate. Parent initiated ego-involving climate was not significantly correlated with any of the engagement or flow variables. Multiple regression analysis showed that parental task-involving climate significantly contributed to athletes’ confidence, dedication, vigour, enthusiasm, global engagement, and dispositional flow.
Schmidt et al 2019 [114]	Investigate the relationship between sport engagement and alcohol consumption.	362 Argentinian athletes in teams sports	Argentinian version of AEQ [108]. Cuestionario de Identificación de los Trastornos Debidos al Consumo de Alcohol (AUDIT-C) [115].	Athletes consume as much alcohol as non-athletes, but those who invest more energy in their sport have a lower frequency of current and heavy drinking.
Schuster et al. 2016 [116]	Determine the latent structure of psychological questionnaires within the Multidimensional Scale of Sports’ Psychological Talents (MSSPT) and explore the correlations between its dimensions and various sport-related variables.	127 Croatian handball players	Multidimensional Scale of Sports’ Psychological Talents (MSSPT) [116]. Include: Revised Life Orientation Test (LOT-R), Big Five Inventory-10 (BFI-10), AES), and Mental Energy Scale (MES).	12 latent dimensions are revealed after the application of PCA, named: Openness/Agreeableness/ Consciousness, Neuroticism/ Openness and Extraversion/ Agreeableness/ Consciousness (from MBFI), Enthusiasm/ Energy, Dedication and Self-esteem (from AES), Energy as motivator, Energy as strength during errors, Energy as lower pressure and Energy as stable performance (from MES), Optimism/ Happiness and Energy (from OS). Almost all the dimensions in all the questionnaires showed satisfactory validity and reliability. Statistically significant correlations between the dimensions of the MSSPT and relevant variables are very few (6 out of 72), mostly related to handball experience. The number of intercorrelations suggests that some of the psychological characteristics within the MSSPT are mostly positively but weakly correlated.
Scotto et al. 2019 [117]	Examine the relationships between the sport sense of community, athlete burnout, engagement, and motivation in adolescent athletes, using a longitudinal perspective, and both person-centred and variable-centred approaches.	250 French athletes	Sport Sense of Community in Adolescence Questionnaire (SSCAQ) [117]. French versions of: ABQ [118]. BRSQ [119]. UWES [117].	Sense of sport community dimensions (i.e. satisfaction of needs and influence) negatively predicted athlete burnout and controlled motivation, and positively predicted engagement and autonomous motivation six months later. Athlete burnout is associated with negative consequences for athletes’ well-being, whereas engagement reflects a positive state. Sense of sport community refers to the athletes’ relationship with their sport environment. In this study, we provided preliminary evidence for the protective role of the sense of sport community against athlete burnout.
Stolarski et al. 2020 [120]	Develop a Polish version of the Sport Engagement Scale and determine its associations with athletic burnout, competition anxiety, personality traits, declared sport level, and number of training hours.	214 Polish athletes 135 Polish half marathon runners	Polish versions of: SES [120]. ABQ [121]. Short version of International Personality Item Pool (Mini-IPIP) [122].	Athletes with higher levels of engagement reported more hours of training per week. The expectation that high SES scores should be associated with lower burnout was fully supported by the data. Emotional stability (the opposite of neuroticism) and conscientiousness were consistently associated with greater sport engagement. There was a slight positive association between dedication and intellect (after controlling for age and sex, the analogous effect was also significant for general engagement score and vigour). The effect of general SES score on final race result was significant (after controlling for age and sex). When the total score on the SES scale was divided into three subscales, only vigour was a significant predictor of final race score.
Tastan et al. 2023 [123]	Examine the role of athlete engagement in determining self-efficacy among orienteering participants and assess whether engagement and self-efficacy vary by sex.	307 Turkish orienteering athletes	AEQ [48]. Athlete Self-Efficacy Scale (ASES) [124].	Athletes’ engagement differed significantly by sex, but self-efficacy did not differ by sex. Athlete self-efficacy was predicted by the variables of confidence and dedication; the variables of vigour and enthusiasm did not have a significant effect on self-efficacy. The four variables explain approximately 22% of the total variance in self-efficacy. Individuals with high athlete engagement have a high belief in their ability to achieve their desired goal, i.e. their self-efficacy, when their performance is high.
Teng and Wang 2020 [125]	Explore the impact of coaches’ professional and emotional competence on athletes’ psychological engagement.	418 Chinese students athletes	Chinese versions of: Coach professional competency [125]. Coach emotional-healing competency [125]. AEQ [125].	There was an inverted U-shaped relationship between coach professional competence and athlete psychological engagement, and a positive relationship between coach emotional-healing competence and athlete psychological engagement. Coaches’ emotional-healing competence moderated the inverted U-shaped relationship between coaches’ professional competence and athletes’ psychological engagement.
Tian and Sun 2023 [126]	Explore the relationships between self-concept clarity, mental toughness, athlete engagement, and athlete burnout to identify factors influencing athlete burnout in swimmers and to investigate effective strategies for alleviating burnout in this group.	189 Chinese swimmers	Chinese versions of: Self-Concept Clarity Scale (SCCS) [127]. MTI [128]. AEQ [129]. ABQ [62, 130].	A direct effect of self-concept clarity on athlete burnout was negative and significant. The mediating effect of athlete engagement and the chain mediating effect of mental toughness and athlete engagement on athlete burnout were also significant. Mental toughness had no significant mediating effect on the relationship between self-concept clarity and athlete burnout, suggesting that self-concept clarity is related to athlete burnout via mental toughness and athlete engagement.
Waleriańczyk and Stolarski 2022 [131]	Examine the associations between perfectionism, athlete burnout, and engagement	173 Polish athletes	Polish versions of: S-MPS-2 [11]. Performance Perfectionism Scale–Sport (PPS-S) [11]. ABQ [132]. SES [120].	Personal standards perfectionism predicted decreases in all symptoms of burnout, except devaluation, and an increase in only one subscale of engagement - dedication. Evaluative concerns perfectionism predicted increases in two symptoms of burnout - exhaustion and reduced sense of accomplishment and decreases in all subscales of engagement.
Waleriańczyk et al. 2022 [11]	Re-test the hypotheses of the 2 × 2 model for engagement and burnout using various measures of perfectionism.	377 Polish athletes	SES [120]. ABQ [132]. S-MPS-2 [11]. PPS-S [11].	Self-oriented performance perfectionism was a significant positive predictor of all dimensions of engagement, whereas socially prescribed performance perfectionism was a significant negative predictor of all dimensions of engagement. Personal standards was a significant positive predictor of all dimensions of engagement, whereas worry about mistakes was a significant negative predictor of commitment and vigour. Self-oriented performance perfectionism was a significant predictor of devaluation and reduced sense of accomplishment, whereas socially prescribed performance perfectionism was a significant positive predictor of all symptoms of burnout.

### Sample Characteristics of Athlete Engagement Studies

Table 3 presents a detailed description of the selected studies concerning demographic characteristics and the type of practiced sport of the athletes involved. Study sample sizes ranged from 15 to 1188 athletes, with an average of approximately 340 athletes per study. The majority of the studies included athletes from various modalities, both individual and team sports, followed by studies with athletes from team sports modalities. Subsequently, studies on soccer predominated (*n* = 3). Other sports were represented by only one study each and included basketball, dance sport, football, handball, karate, marathon, orienteering, rowing, skiing, swimming, tennis, track and field, and volleyball. The majority of studies included more male than female participants. Nine studies had predominantly female samples [2–4, 9, 36, 69, 102, 112, 125], five studies included only male participants [1, 5, 31, 94, 116], and two studies included only female participants [81, 96]. Sex distribution was not specified in two studies [32, 63].

The average age of the athletes was approximately 19.27 years (*SD* = 3.75; with a range between 10 and 67 years). In four studies, no information about age was disclosed [12, 47, 63, 89], and in one study [28], only the minimum and maximum age range was provided. Generally, the studies were conducted with adolescent and/or young populations (12–30 years old). Data on other contextual factors in sport, such as the level of competition or training load, were not disclosed in all the articles, making the analysis more difficult. When more information is provided, it can be observed that the studies generally select athletes from different sporting levels (50%). Regarding training load, nearly half of the studies provide data on the hours dedicated to training weekly or even the number of weekly sessions along with their average duration. In this way, an average of 8.75 h of training per week was observed (with a range between 4.28 and 17.76 h/week).

**Table 3:** Description Principal characteristics of the sample

Study	*n*	Female	Male	M_age_	SD_age_	Range_age_	Sport
[28]	310	105	205	-	-	11–18	Basketball
[12]	71	27	44	-	-	-	Track and field
[31]	93	0	93	18.6	1.98	-	Team sports
[32]	232	-	-	14.68	1.14	-	Volleyball
[36]	260	150	110	13.53	1.27	11–16	Soccer
[38]	509	132	377	17.36	4.58	-	Multisport
[42]	426	114	214	17.55	4.25	-	-
[39]	1157	423	734	17.89	3.58	13–28	Multisport*
[8]	1011	506	505	18.09	5.55	-	Multisport*
[1]	210	0	210	19.7	1.4	18–24	Football
[47]	834	242	592	-	-	-	Multisport**
[49]	500	130	370	17.39	4.6	12–29	Multisport
[51]	326	150	176	19.63	6.51	14–26	Team sports
[7]	287	102	185	19.63	2.53	14–26	Team sports
[5]	240	0	240	23.75	4.29	15–38	Soccer
[59]	352	145	207	18.83	4.27	-	Team sports
[63]	290	-	-	-	-	-	-
[64]	150	65	85	15.73	1.26	14–19	Multisport
[69]	297 222 211	159 124 72	127 98 139	15.16 16.01 18.09	1.93 2.68 1.38	- - -	Swimming Multisport Multisport
[9]	201	121	80	22.92	-	14–61	Multisport
[75]	265	94	171	18.81	3.73	15–30	Tennis
[10]	222	124	98	16.01	2.68	-	Multisport
[48]	201	87	114	23.32	2.84	-	Multisport
[81]	258	258	0	19.82	3.89	17–24	Multisport*
[6]	992 465	425 201	567 264	13.5 13	0 0	- -	Multisport Multisport
[86]	100	18	82	30.21	10.64	17–65	Karate and soccer
[89]	242	122	120	-	-	-	Dance sport
[4]	15	7	7	24.2	-	18–45	Multisport
[3]	382 201 343	180 121 183	202 80 160	25.9 22.9 24.5	8.1 7.2 7.7	-	Multisport
[91]	367	135	232	16.18	2.02	-	Multisport
[94]	227	0	227	23.36	3.63	18–32	Soccer
[77]	771 414	157 78	614 336	20.2 17.2	6.28 4.63	- -	Multisport Multisport
[96]	37	37	0	19.3	1.18	-	Rowing
[2]	176	141	35	15.87	1.74	13–19	Multisport
[100]	177	78	99	16.36	3.79	12–31	Multisport
[102]	192	97	95	17.17	1.04	16–20	Skiing
[104]	517	201	316	16.01	1.85	12–20	Basketball and volleyball
[108]	1188	521	667	15.92	2.5	10–26	Multisport
[112]	180	98	82	17.48	3.39	-	Multisport
[114]	362	155	207	18.2	3	15–29	Team sports
[116]	127	0	127	13.88	4.14	-	Handball
[117]	250	103	147	15.65	1.6	-	Multisport
[120]	214 135	91 50	123 85	28.26 36.67	11.09 9.04	18–67 19–67	Multisport Marathon
[123]	307	140	167	12.44	1.2	-	Orienteering
[125]	418	243	175	19.44	3.26	17–25	Multisport
[126]	189	113	76	13.71	2.57	9–25	Swimming
[131]	173	85	88	27.47	6.11	18–39	Multisport
[11]	377	201	176	26.13	6.01	18–40	Multisport

n = number of participants; *M*_age_ = mean age; *SD*_age_ = standard deviation of age

*predominance of team sports

** students in sports sciences

### Instruments

The majority of the reviewed studies employed questionnaires to assess engagement in athletes. The AEQ was the most frequently used instrument, appearing in 37 studies and representing 79.16% of the total. This questionnaire is widely recognized for its efficacy in measuring athletes’ specific engagement in their sports activities and was developed by Lonsdale and colleagues in 2007 [3, 4]. The scale comprises 16 items, grouped into four dimensions, with four items in each dimension. The dimensions are as follows: confidence defined as the ability to excel and achieve results (‘I believe I am capable of accomplishing my goals in sport’), dedication characterised by a sense of purpose, pride, and challenge (‘I am determined to achieve my goals in sport’), vigour defined by its high energy levels, and which leads to resilience and effort during challenges (‘I feel really alive when I participate in my sport’), and enthusiasm that arises when an individual is engaged in a task that brings about a sense of satisfaction, characterised by a heightened level of focus, efficiency and deep engagement in the activity (‘I feel excited about my sport’). Each dimension is measured by means of four items using a 5-point Likert-type response from 1 (Almost never) to 5 (Almost always). This scoping review collates diverse adaptations and translations of the AEQ to evaluate its validity and reliability in disparate cultural contexts. The AEQ has been translated into and validated in several cultures, including Argentinian [57], Chinese [129], Croatian [12], Greek [83], Portuguese [77], Spanish [38, 42], and Turkish [48]. On the other hand, the SES (also known as the Athlete Engagement Scale; AES) was employed in six studies, constituting 12.5% of the total. SES is composed of 12 items distributed between three factors: vigour (four items), dedication (three items), and absorption (five items). It is responded to by a Likert scale ranging from 1 (Rarely) to 7 (Nearly always). Examples of items corresponding to three factors are: ‘I am strong and vigorous in my sport activity’ (vigour); ‘I feel inspired whilst carrying out my sport activity’ (dedication); ‘Time flies when I am training or competing’ (absorption). This instrument has been adapted for use in the sport context from the Utrecht Work Engagement Scale (UWES), which is a more general measure of engagement across a range of occupational contexts. In addition to the original version [5], this scoping review has identified the validation of the SES in the Turkish and Polish sports contexts [30, 120]. The UWES was employed by researchers as a measure of sports engagement in three studies (6.25%) in its French and Portuguese versions [86, 91, 117]. The final questionnaire employed was the SpEI in one study, representing 2.08% of the total research. Additionally, interviews were utilised in one study (2.08%). The use of interviews allows for a qualitative approach that facilitates a more profound and comprehensive understanding of the engagement levels of athletes.

### Correlated or Exposure Factors of Athlete Engagement

Across the studies, 41 correlates of athlete engagement were identified. As illustrated in Table 4, these variables have been examined in relation to athlete engagement as antecedents, consequences, mediators/moderators, or correlates. Five demographic variables were identified as antecedents of engagement or its dimensions (age, level of education, family income, gender/sex, and area of residence). Three sport-related variables were examined as antecedents including performance, which was also analysed as a consequence and as a mediator. The remaining 33 variables were of a psychological nature.

**Table 4:** Exposure factors of athlete engagement

Variable	Antecedents	Consequences	Mediator/ moderator	Related variable
Age	[28]			
Areas of worklife	[1]			
Athletes sports success/performance	[12]	[63] [89]	[63]	
Athletes’ and coaches’ perceptions	[104]			
Athletic identity				[12]
Autonomy support	[100]			
Basic psychological needs	[39] [8] [9] [91] [94] [102]		[10]	[108]
Burnout		[1] [49]	[1]	[31] [42] [8] [59] [48] [6] [4] [108] [120]
Coach emotional-healing competency	[125]			
Coach leadership style	[28] [31]			
Coach professional competency	[125]			
Coach-athlete relationship	[31] [7] [96]			
Coaching behavior			[63]	
Cohesion	[51]			
Dispositional flow		[9]		
Duration of playing	[28]			
Education level	[28] [12]			
Effect of the partnership	[89]			
Enjoyment				[108]
Family income	[28]			
Sex	[28] [12]			
Gratitude	[59]			
Mental energy				[116]
Mental toughness	[32]	[75]	[51] [126]	
Motivation	[49]		[8] [39] [102]	[108]
Motivational climate	[36] [64] [104]		[64]	
Negative physical self			[32]	
Observed behavior in training	[104]			
Optimism				[116]
Parent-initiated motivational climate	[111]			
Perceptions of benefits and risks of sport	[86]			
Perfectionism	[10] [131] [11] [69]			
Personality				[120] [116]
Psychological collectivism			[51]	
Quality of life		[86]		
Residential area	[28]			
Self-concept clarity	[126]			
Social support	[2]			
Sport sense of community	[117]			
Subjective well being			[32]	
Thriving			[7]	

Most research has focused on explaining antecedent variables of engagement. Basic psychological needs and perfectionism were the two most studied antecedents (six studies), followed by motivational climate (four studies linking motivation and climate motivation; three when considering climate motivation alone) and the coach-athlete relationship (three studies).

In terms of consequences, the researched variables were athletes’ sports success/competitive performance, burnout, dispositional flow, mental toughness, and quality of life. Performance and burnout were the most studied consequences (two studies).

Motivation is the most studied mediator (three studies, or four if motivational climate is included), with mental toughness being the second most studied (two studies). Other mediator/moderator variables include athletic performance, basic psychological needs, burnout, coaching behaviour, negative physical self-concept, psychological collectivism, subjective well-being, and thriving.

The simultaneous relationship between engagement and other variables has also been studied, such as burnout (nine studies), personality (two studies), athletic identity, basic psychological needs, enjoyment, mental energy, motivation, and optimism (all of them with a single study).

This analysis revealed that burnout represents the most extensively studied variable in the context of athlete engagement. This underscores the significance of burnout as both a consequence [1, 49], mediator [1] and as a variable that is simultaneously related to engagement [4, 8, 31, 42, 48, 59, 120]. It is also noteworthy that a considerable body of research has examined engagement as a precursor or mediator of other variables. These include athletic performance [63, 120], mental toughness [75], alcohol consumption [114], self-efficacy [123], physical self-worth [81] and resilience [100].

Although most studies examined specific antecedents and correlates of athlete engagement using quantitative methods, one foundational qualitative study [3] explored athletes’ lived experiences of engagement. This investigation identified four core dimensions which later informed the development of the AEQ. These dimensions align with several of the key variables reported in the quantitative literature.

## Discussion

The present study aimed to investigate which factors within the sporting context are related to athlete engagement, based on empirical evidence. To this end, a scoping review was conducted, with a total of 48 studies published between 2007 and 2024 selected for analysis. The majority of the selected studies were quantitative and cross-sectional. The samples were predominantly composed of males and females engaged in a diverse range of sports and at varying levels of competition. The majority of studies employed questionnaires, such as the AEQ, to measure engagement. These findings are consistent with those of previous studies which have indicated that the AEQ is an effective and validated tool for the assessment of athlete engagement. Notably, the development of the AEQ was grounded in an early qualitative investigation that provided critical conceptual insight into the engagement construct [3]. By exploring elite athletes’ lived experiences, this study identified the core dimensions of engagement. These dimensions became the basis for the AEQ and helped distinguish athlete engagement from related constructs such as enjoyment, commitment, or satisfaction. While qualitative research remains scarce in this field, its contribution is essential for capturing the subjective and contextual complexity of engagement experiences, an aspect that complements and enriches quantitative findings.

### Burnout: the Most Extensively Studied Correlate

In the sports context, burnout is typically defined as a psychological syndrome comprising emotional and physical exhaustion, a reduced sense of accomplishment, and sport devaluation [133]. This syndrome emerged as the most frequently investigated variable in relation to athlete engagement, appearing in 12 studies across different relational categories. This extensive research attention reflects the theoretical importance of understanding the burnout-engagement relationship in sport psychology.

Two primary theoretical perspectives exist regarding this relationship. The initial conceptualization posits that engagement and burnout are diametrically opposed phenomena on a single continuum. In contrast, the alternative approach suggests they are distinct but inversely correlated constructs that exhibit more similarities than differences [134]. Our review found evidence supporting both perspectives, with most validation studies of engagement measures including burnout analyses [3, 4, 42, 48, 108].

Following the independent constructs approach, several studies employed dual models in their research, analyzing the same predictor/mediator variables with burnout as the dependent variable in one model and engagement in the other. This methodological approach was particularly evident in perfectionism research [10, 11, 131] and basic psychological needs studies [91], allowing researchers to examine how the same factors differentially influence both constructs.

Research demonstrates that this relationship is bidirectional and complex. Individuals experiencing burnout due to inability to cope with sport demands show notable declines in engagement levels [31]. Conversely, burnout can act as both a barrier to maintaining high engagement over time and as a consequence of insufficient engagement [1]. Cross-lagged analyses revealed that engagement at time 1 significantly and negatively predicted burnout at time 2, while burnout at time 1 also negatively predicted engagement at time 2 [59].

### Self-Determination Theory: Basic Psychological Needs and Motivational Regulations

Self-determination theory (SDT) is a broad framework for understanding human motivation, within which Basic Psychological Needs Theory and Organismic Integration Theory are particularly relevant to this review. The first posits that the satisfaction of autonomy, competence, and relatedness is essential for optimal functioning and well-being, while the second describes a continuum of motivational regulation ranging from extrinsic to intrinsic motivation [135, 136].

Basic psychological needs emerged as the second most studied antecedent of engagement (six studies), reflecting the central role of SDT in understanding athlete engagement. The consistent research attention suggests that satisfaction of autonomy, competence, and relatedness represents a fundamental aspect of athlete engagement.

Research consistently demonstrates that when athletes feel in control of their activities (autonomy), competent in their skills (competence), and meaningfully connected with others (relatedness), their engagement levels increase significantly. These needs show both direct positive effects on engagement and indirect effects mediated through self-determined motivation [8, 39]. Need satisfaction and thwarting also function as mediators between perfectionism and engagement, with need satisfaction promoting engagement and need thwarting reducing it [10].

The four motivational regulations (intrinsic, identified, integrated, and external regulation) function as mediators in the relationship between basic psychological needs and engagement [102]. High levels of self-motivation consistently correlate with increased athlete engagement [39], though this relationship may be moderated by how motivation is conceptualized across studies [8]. Additionally, research using Vallerand’s global motivation index [137] demonstrated that this comprehensive measure was not linked to burnout, but was linked to engagement. This means that engagement may serve as a protective factor against burnout [49].

### Perfectionism: Adaptive Versus Maladaptive Dimensions

In the perfectionism literature, higher-order dimensions are often referred to as perfectionistic strivings—characterized by the pursuit of high personal standards—and perfectionistic concerns—marked by fear of mistakes and negative evaluation [138]. Perfectionism research reveals complex interactive effects on athlete engagement across multiple studies [10, 11, 69, 131]. The highest engagement levels associate with pure personal standards perfectionism (high personal standards/low evaluative concerns), while the lowest levels associate with pure evaluative concerns perfectionism (low personal standards/high evaluative concerns) [69].

Perfectionism influences engagement through basic psychological needs satisfaction and thwarting [10]. Perfectionistic concerns negatively correlate with engagement through need satisfaction, while perfectionistic striving shows positive relationships with engagement via need satisfaction. These findings highlight the importance of distinguishing between adaptive and maladaptive perfectionism dimensions when examining their relationships with athlete engagement and burnout [11, 131].

### Motivational Climate: Environmental Influences

Motivational climate refers to the perceived emphasis in the sporting environment, which can be mastery-oriented—focusing on personal improvement and effort—or performance-oriented—emphasizing comparison and competition with others [139, 140].

Motivational climate research demonstrates that environmental factors significantly influence engagement. Mastery climates—emphasizing task mastery and personal improvement—are consistently associated with higher overall engagement levels. In contrast, performance climates—focused on competition and comparison—have more complex effects. While they can negatively impact engagement by increasing pressure and stress, they have also been shown to positively influence certain cognitive aspects of engagement, such as mental focus and task concentration [36].

Further research examining parent-, coach-, and peer-initiated motivational climates reveals differential impacts on athlete engagement [64]. Among these, coach-created climates consistently emerge as the strongest predictors, particularly when they promote autonomy and task involvement. Interestingly, the influence of mastery climates appears weaker when initiated by parents, highlighting the pivotal role of coaches and peers in shaping engagement.

Building on these findings, Achievement Goal Theory (AGT) offers a broader theoretical framework to explain how motivational climates influence engagement. Studies comparing empowering and disempowering climates further underscore the role of environmental factors in shaping athletes’ behavior, emotions, and cognition [104]. Once again, athletes’ perceptions of coach-created climates—especially those fostering autonomy and task involvement—have been identified as particularly strong predictors of engagement.

### Coach-Athlete Relationship: Interpersonal Foundations

The coach–athlete relationship, in Jowett’s 3 + 1Cs model, comprises closeness (affective/emotional bonds), commitment (cognitive intentions to maintain the partnership), and complementarity (behavioral cooperation), which together underpin co-orientation—shared perceptions of goals, roles, and expectations [141, 142]. Positive and supportive coach-athlete relationships consistently associate with higher engagement levels [7, 31], with closeness identified as a significant predictor of engagement across competitive seasons [96].

### Limitations

Several limitations should be acknowledged when interpreting these findings. First, the predominance of cross-sectional studies (87.5%) limits our understanding of how athlete engagement develops and changes over time. Second, most research focused on adolescent and young adult populations, potentially limiting generalizability to other age groups, particularly older athletes or those in late-career phases. Third, the majority of studies employed quantitative methods, with only one qualitative investigation, restricting deeper understanding of athletes’ lived experiences of engagement. Finally, the reliance on self-report measures (primarily AEQ) may introduce common method bias and limit the comprehensiveness of engagement assessment.

### Future Research

Our synthesis reveals several critical areas requiring future investigation. Longitudinal studies are needed to understand how engagement evolves across athletic careers and identify critical periods for intervention. Qualitative research should explore athletes’ subjective experiences of engagement to complement quantitative findings and inform theory development. Intervention studies testing engagement-enhancing programs based on identified antecedents (e.g., basic psychological needs satisfaction, positive coach-athlete relationships) are essential for translating research into practice. Multi-method approaches combining self-report, behavioral, and physiological indicators could provide more comprehensive engagement assessment. Finally, theoretical model development integrating the 33 identified correlates into comprehensive frameworks represents a critical next step for the field.

### Practical Implications

These findings offer concrete guidance for sports practitioners seeking to enhance athlete engagement. For coaches, prioritizing the development of positive coach-athlete relationships, creating mastery-oriented motivational climates, and supporting athletes’ autonomy, competence, and relatedness needs emerge as evidence-based strategies. Training programs should incorporate burnout prevention strategies, given its strong negative relationship with engagement, including adequate recovery periods and monitoring of psychological well-being. For sport psychologists, addressing maladaptive perfectionism remains a key intervention target. While certain elements often described as “adaptive” perfectionism may be linked to high personal standards and energising qualities, emerging evidence indicates that they can also undermine well-being and should therefore be approached with caution [143]. Organizational approaches should focus on creating supportive environments that satisfy basic psychological needs and promote intrinsic motivation. Parent education programs teaching supportive motivational climate creation could complement coach-focused interventions. These evidence-based recommendations provide a foundation for developing comprehensive engagement-enhancement programs across various sporting contexts.

## Conclusions

Athlete engagement has emerged as a central construct in the field of sport psychology, offering a valuable perspective for understanding how athletes maintain and enhance their performance over time. Notwithstanding the considerable progress that has been made in the investigation of this phenomenon, the extant literature reveals several significant shortcomings. For instance, although numerous factors affecting engagement have been identified, the interplay between them and their long-term consequences remain poorly understood. Accordingly, this scoping review has identified a comprehensive range of factors related to athlete engagement, thereby establishing a foundation for future research and practical applications in sport. These will facilitate the design of training programs that enhance engagement.

## Data Availability

All data generated or analyzed during this study are included in this published article and its supplementary information files.

## Abbreviations

AES: Athlete Engagement Scale
AEQ: Athlete Engagement Questionnaire
AGT: Achievement Goal Theory
APA: American Psychological Association
CNKI: China National Knowledge Infrastructure
EBSCO: Elton B. Stephens Company
ERIC: Education Resource Information Center
JBI: Joanna Briggs Institute
M: Mean
n: Number of participants
PECOS: Population, Exposure, Comparison, Outcome, Study design
PICOS: Population, Intervention, Comparison, Outcome, Study design
PRISMA: Preferred Reporting Items for Systematic reviews and Meta-Analyses
RCTs: Randomised controlled trials
SD: Standard deviation
SDT: Self-Determination Theory
SES: Sport Engagement Scale
SpEI: Sport Engagement Instrument
T1: First time point
T2: Second time point
